# Biomechanical and Aerodynamic Modulation for Sinonasal Homeostasis in Craniofacial Orthopedics: A Comprehensive Review of RAMPA Therapy

**DOI:** 10.3390/biomimetics11070494

**Published:** 2026-07-14

**Authors:** Yasushi Mitani, Yuko Okai-Kojima, Mohammad Moshfeghi, Bumkyoo Choi, Yoshiya Hashimoto

**Affiliations:** 1 Private Practice, Codomo Clinic, Tokyo 180-0004, Japan; mitani@trust.ocn.ne.jp; 2 Private Practice, Children and Women Dental Clinic, Tokyo 106-0046, Japan; yukoyukono@gmail.com; 3 Department of Mechanical Engineering, Sogang University, 35 Baekbeom ro, Mapogu, Seoul 04107, Republic of Korea; mohammad.moshfeghi@gmail.com; 4 Department of Biomaterial, Osaka Dental University, Hirakata 573-1121, Japan; yoshiya@cc.osaka-dent.ac.jp

**Keywords:** RAMPA therapy, mechanotransduction, Bone Morphogenetic Protein-2 (BMP-2), computational fluid dynamics (CFD), shear-thinning mucus rheology, sinonasal homeostasis, craniofacial remodeling

## Abstract

Background: Maxillary hypoplasia and skeletal Class III malocclusion are deeply intertwined with upper airway constriction and paranasal sinus dysfunction. Conventional orthopedic interventions often struggle to achieve true 3D skeletal translation without inducing undesirable rotational side effects. The Right Angle Maxillary Protraction Appliance (RAMPA) therapy offers a biomimetic and mechanotherapeutic approach, focusing on anterosuperior protraction to restore both structural harmony and respiratory function. Methods: Structured as a comprehensive narrative review, this feature paper thoroughly reviews the multi-disciplinary evidence supporting RAMPA therapy by synthesizing our group’s recent computational (FEM, CFD) and clinical findings alongside the broader literature. We examine Finite Element Method (FEM) simulations detailing sutural mechanotransduction and osteogenic “BMP-2 Trigger Zones”, Computational Fluid Dynamics (CFD) utilizing shear-thinning rheological models for two-phase air–mucus interactions, and large-cohort CBCT and Coben analyses quantifying longitudinal growth. Results: FEM studies confirm that RAMPA, especially when combined with intraoral devices (e.g., gHu-1, VomPress, Hybrid), achieves predictable anterosuperior displacement and concentrates tensile stress to levels hypothesized to stimulate molecular bone remodeling. CFD simulations reveal that this precise skeletal remodeling optimizes wall shear stress (WSS) and theoretically facilitates paranasal mucus clearance via enhanced suction and shear-thinning effects. Clinically, RAMPA induces a 1.2-fold acceleration in natural sinonasal growth velocity. Furthermore, volumetric gains are distinctively pronounced in patients with pre-existing empyema (61.2% increase) compared to those with clear sinuses (18% increase), indicating rapid pathophysiological obstruction relief. Conclusions: By integrating controlled biomechanical forces with fluid-dynamic airway optimization, RAMPA therapy acts as a mechanotherapeutic modulator. It bridges the gap between mechanical intervention, molecular signaling, and physiological homeostasis, offering a comprehensive paradigm for pediatric craniofacial and respiratory restoration.

## 1. Introduction

### 1.1. Craniofacial and Sinonasal Growth as Determinants of Pediatric Airway Function

Craniofacial growth and pediatric airway function operate as an interdependent biomechanical system. Foundational paradigms and subsequent clinical evidence have consistently demonstrated that upper airway obstruction—often associated with adenoid hypertrophy and mouth-breathing—is strongly linked to altered craniofacial morphology, including a steep mandibular plane, mandibular retrognathia, and reduced nasopharyngeal volume [1,2,3,4,5,6,7,8,9]. Because airway dimensions and structural configurations evolve dynamically throughout childhood and adolescence [10,11,12], optimizing pediatric respiratory function requires a comprehensive 3D structural approach. Furthermore, sinonasal development and inflammatory conditions, such as allergic rhinitis and chronic rhinosinusitis (CRS), profoundly influence midfacial growth and airway stability [13,14,15,16,17,18], highlighting the profound complexity and heterogeneity of craniofacial–airway relationships in contemporary interdisciplinary research [19,20].

### 1.2. Airway Biomechanics, Aerodynamics and Mucus Physiology

The airway relies on a highly coordinated aerodynamic apparatus to maintain pulmonary sterility via mucociliary clearance [21,22,23,24], and this topic has been a point of interest for many researchers. For instance, Button et al. [25], have reframed this process through the gel-on-brush model, providing a biophysical explanation for how tethered mucins form a dense periciliary glycocalyx to regulate transport. At the macroscopic level, mucus functions as a dynamic viscoelastic hydrogel whose tissue-specific mechanical properties govern deformation, transport, and clearance across mucosal surfaces, thereby contributing to epithelial homeostasis [26]. Mucus solids concentration governs viscoelasticity and transport, with a critical gel transition near 4% weight percent solids that can impair mucociliary clearance and alter airway barrier function [27].

Airway mucus provides a critical defence barrier in which the gel-forming mucins MUC5B and MUC5AC constitute major structural components and contribute to mucociliary clearance [23]. Effective mucus transport depends on the integrity of the periciliary layer [25], while mucus viscoelasticity and transport efficiency are strongly governed by solids concentration, airway surface hydration, and mucin composition [27,28,29,30,31,32]. MUC5B predominates in healthy airways, whereas increased MUC5AC is particularly associated with inflammatory and obstructive airway disease [32,33,34,35].

As mucus becomes hyperconcentrated, its osmotic pressure and viscoelasticity increase, promoting periciliary-layer compression, impaired mucociliary transport, and mucus stasis [36,37,38,39]. These concentration-dependent changes also increase mucus adhesion, cohesion, and viscous energy dissipation, thereby reducing the efficiency of airflow-driven clearance [40].

### 1.3. Orthopedic Modulation of Airway Structure

Orthopedic modulation of the maxilla has long been recognized as a central mechanism for altering upper airway structure. Decades of research, ranging from early clinical applications of rapid maxillary expansion (RME) to advanced finite element method (FEM) and computational fluid dynamics (CFD) analyses, confirm that conventional expansion enlarges the nasal cavity, increases nasopharyngeal volume, and reduces airflow resistance [41,42,43,44,45,46]. The evolution of skeletal anchorage systems, such as MARPE, further improved expansion by distributing stress more uniformly across the palate [47,48]. Computational models utilizing validated material properties [49,50,51,52] have provided additional support for these mechanisms, highlighting age-dependent sutural responsiveness [42,46,53,54] and concurrent soft-tissue adaptations [55,56].

However, while these conventional modalities successfully provide transverse (lateral) expansion, they frequently struggle to control sagittal and vertical force vectors, resulting in downward displacement and undesirable rotational side effects.

Building upon these foundational principles, this review frames RAMPA therapy not merely as an orthodontic tool but as a mechanotherapeutic modulator. In this context, we define a ‘mechanotherapeutic modulator’ as a clinical intervention that applies targeted, controlled biomechanical forces designed to influence biological and physiological responses—specifically, aiming to stimulate sutural bone remodeling via cellular mechanotransduction and optimizing internal respiratory aerodynamics through structural expansion. To ensure full academic transparency, it should be noted that the authors of this review have been integrally involved in the initial development, continuous clinical refinement, and computational investigation of RAMPA therapy. Consequently, this feature paper inherently synthesizes our group’s decade-long interdisciplinary research alongside the broader literature to present a comprehensive mechanotherapeutic framework.

### 1.4. Biomechanical Efficacy of RAMPA in Orthopedic Maxillary Protraction

**Figure 1:**
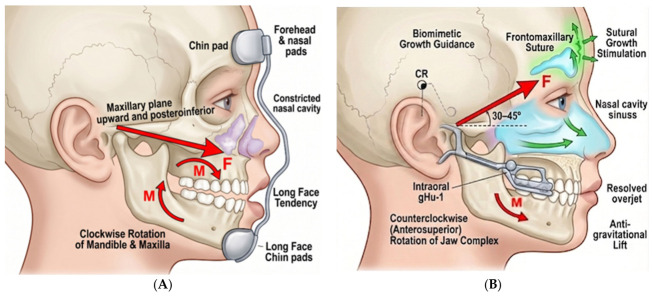
Conceptual Diagram of RAMPA Therapy’s Biomimetic Approach and Comparison to Conventional Facemask: (**A**) Conventional Facemask Therapy, (**B**) RAMPA Therapy.

To overcome the biomechanical limitations of conventional devices, the Right Angle Maxillary Protraction Appliance (RAMPA) was developed as a structural alternative. While traditional facemask therapy (Figure 1A) typically applies orthopedic forces—quantitatively ranging from 300 to 500 g per side—its traction vector is generally directed anteroinferiorly (downward) relative to the skull. This inadvertently induces undesirable rotational side effects; the literature consistently reports adverse outcomes such as a 1° to 3° undesired backward (counterclockwise) rotation of the maxilla and significant extrusion of the upper molars.

By contrast, the RAMPA system (Figure 1B) utilizes its rigidly anchored extraoral frame to convert individualized elastic forces into a definitive, net anterosuperior protraction vector [57]. This precise vector control generates a biomechanically desirable forward rotation, elevating the center of rotation and providing an anti-gravitational lift to the entire maxillomandibular complex [58,59,60]. By directing forces anterosuperiorly, this mechanotherapy actively stimulates circummaxillary sutures, guiding biomimetic structural remodeling rather than mere downward displacement. Clinical, cephalometric, and CBCT volumetric studies, including integrations with intraoral devices, suggest that this specific anterosuperior trajectory effectively increases sinonasal volume, which is hypothesized to subsequently improve airflow dynamics, pressure gradients, and mucus clearance [61,62,63,64].

Collectively, the evidence indicates that RAMPA offers a promising structural alternative to existing orthodontic methods by delivering predictable anterosuperior maxillary advancement and significant sinonasal volumetric gains. Its integrated biomechanical and aerodynamic rationale positions RAMPA as a physiologically aligned therapeutic option for pediatric airway enhancement.

Despite the growing body of evidence, a critical gap remains in the literature regarding a unified framework that seamlessly connects orthopedic mechanical forces with molecular biological responses and aerodynamic fluid mechanics. To fill this gap, the original contribution of this feature paper is to provide a comprehensive review that integrates nearly a decade of interdisciplinary research, ranging from foundational bone physiology to advanced computational modeling (FEM and CFD) and clinical longitudinal studies. Our objective is to establish a cohesive scientific framework that elucidates how RAMPA therapy may optimize craniofacial harmony, sinonasal geometry, and respiratory function, thereby positioning it as a physiologically aligned paradigm in pediatric airway management.

## 2. Description of the RAMPA System and Mechanotherapy Principle

**Figure 2:**
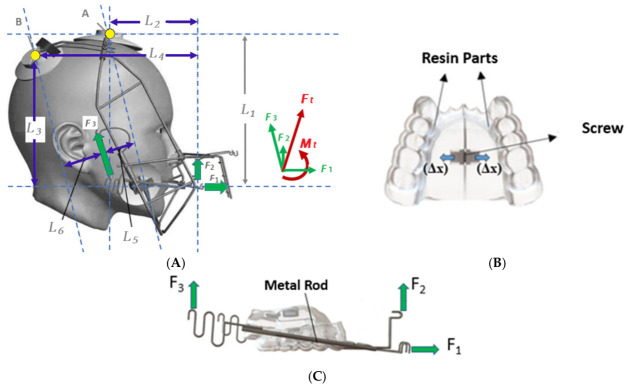
Overview of the RAMPA appliance system and mechanotherapy setup: (**A**) Extraoral device, RAMPA, (**B**) Intraoral device, (**C**) Intraoral device connected with bow.

To realize the theoretical anterosuperior force vector hypothesized to stimulate physiological circummaxillary suture growth, the RAMPA system utilizes a specialized, extraoral framework combined with patient-specific intraoral appliances. Figure 2 provides a comprehensive overview of the physical components and the therapeutic setup.

The extraoral component, RAMPA (Figure 2A), is a robust, rigid metallic structure engineered to establish a stable reference frame relative to the calvaria. The functional hallmark of this extraoral unit is its customizable force delivery system, designed to exert vectors at precise perpendicular or obtuse angles relative to the occlusal plane. The intraoral appliance consists of acrylic resin and the screw for activation as shown in Figure 2B. It is connected to the bow for RAMPA System configuration and applied to patients as shown in Figure 2C. The applied forces from rubber bands in RAMPA, i.e., the horizontal force F1, the front-vertical force F2, and the rear force F3, are shown in Figure 2A. Each force is applied symmetrically to both sides with respect to the center line of the nose.

The strategic integration of these specific force components (F1, F2, and F3) is engineered to synthesize a net biomechanical vector that fundamentally differs from conventional orthopedic treatments. Figure 1 conceptually illustrates this biomechanical synergy and its clinical implications. While traditional facemask therapy (Figure 1A) typically applies orthopedic forces—quantitatively ranging from 300 to 500 g per side—its traction vector is generally directed anteroinferiorly (downward) relative to the skull. This inadvertently induces undesirable rotational side effects; literature consistently reports adverse outcomes such as a 1° to 3° undesired backward (counterclockwise) rotation of the maxilla, significant extrusion of the upper molars, and a subsequent clockwise rotational moment of the mandible. By contrast, the Right Angle Maxillary Protraction Appliance (RAMPA) therapy is structurally designed to address these limitations. As depicted in Figure 1B, the RAMPA system utilizes its rigidly anchored extraoral frame to convert individualized elastic forces into a controlled, net anterosuperior protraction vector. This vector control is mechanically designed to generate a forward rotation, aiming to provide an anti-gravitational lift to the entire maxillomandibular complex. By directing forces anterosuperiorly, this mechanotherapy is theorized to actively stimulate the frontomaxillary and circummaxillary sutures, guiding true three-dimensional biomimetic structural remodeling rather than mere downward displacement.

## 3. Biomechanical Foundations: Finite Element Modeling (FEM)

The proposed biomechanical mechanism of RAMPA therapy lies in its ability to modulate mechanical stress across the craniofacial sutures. A series of high-fidelity FEM investigations have been utilized to model and estimate these biomechanical interactions.

### 3.1. Anterosuperior Protraction and Center of Rotation

**Figure 3:**
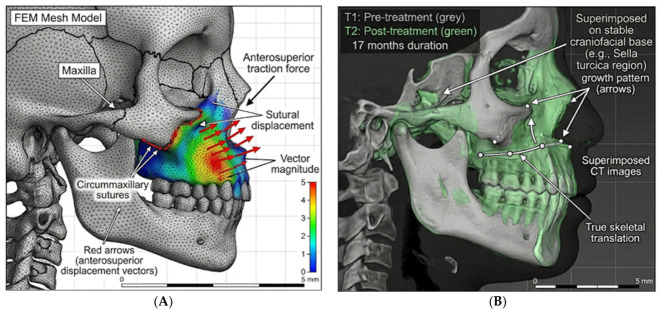
Comparison of FEM simulation results and clinical case of anterosuperior maxilla displacement by RAMPA appliance. (**A**) FEM Simulation: Visualizes the anterosuperior displacement vectors (red arrows) and displacement contours appearing on the maxilla during RAMPA appliance wear. Displacement magnitude increases as the color progresses from blue (low displacement) to red (high displacement). (**B**) Clinical Case: Superimposes 3D CT images of the same patient before treatment (T1, grey) and after 17 months of treatment (T2, green), confirming the same anterosuperior growth pattern as the simulation at the actual skeletal level.

Traditional maxillary protraction frequently forces the maxilla to move in a downward and forward trajectory. FEM analyses suggest that the RAMPA extraoral appliance is designed to counteract this downward vector. When subjected to anterosuperior force vectors, RAMPA is predicted to shift the center of rotation toward the superior-posterior cranial base. This shift theoretically translates into a forward and upward bodily movement of the maxillopalatal complex, minimizing the downward tipping of the palatal plane and potentially mitigating the typical clockwise rotation of the mandible. These simulated biomechanical dynamics are visually illustrated in Figure 3. The FEM simulation (Figure 3A) explicitly maps the theoretical anterosuperior displacement vectors across the maxilla. Crucially, this theoretical model aligns with observational clinical evidence; 3D CT superimpositions (Figure 3B) over a 17-month treatment period exhibit a skeletal translation that mirrors the predicted anterosuperior growth pattern.

### 3.2. Synergies with Intraoral Appliances

The integration of RAMPA with specific intraoral devices—such as the gHu-1, VomPress, and Hybrid appliances (summarized in Table 1)—is designed to further influence this displacement. Simulations of RAMPA combined with the gHu-1 semi-rapid maxillary expansion (sRME) device suggested that the extraoral upward pull does not mechanically interfere with the lateral expansion generated by the intraoral screw. Instead, it is predicted to shift mid-palatal suture displacement from a slight postero-inferior drift into a more favorable anterosuperior trajectory. Furthermore, combinations with the VomPress and novel Hybrid intraoral appliances have yielded promising observational outcomes. Both FEA and supporting clinical case reports of growing patients (e.g., a 7-year-old female treated over 17 months) indicated that the Hybrid/RAMPA combination was associated with a counterclockwise mandibular rotation (−1.0 degree) and a decrease in the RAMUS angle (2.9 degrees), suggesting a potential role in mitigating long-face tendencies and supporting improvements in overall facial profile and cervical posture.

### 3.3. Predictability Across Suture Maturation

Because biological tissue properties vary by age and individual, the impact of suture stiffness (Young’s modulus) on treatment outcomes is a critical clinical variable. The fundamental assumption of these FEM studies is that while the maxillary bones exhibit linear, homogeneous, and isotropic elastic behavior, the circummaxillary sutures are highly variable. To reflect this, specific sutural moduli were selected to represent progressive stages of pediatric sutural maturation: 30 MPa simulates the highly flexible sutures of early mixed dentition (approx. 7–9 years), 50 MPa reflects intermediate maturation (approx. 10–12 years), and 80 MPa represents the more rigid, interlocked sutures typical of early adolescence (approx. 13–15 years or older) [60]. The FEM simulations with varied sutural moduli (30, 50, and 80 MPa) against RAMPA forces indicated that structural displacement and von Mises stresses possess a predictable, linear relationship with suture stiffness. Unlike rigid models that exhibit erratic stress concentrations, flexible physiological suture models suggest that RAMPA theoretically maintains a consistent anterosuperior protraction pattern across a broad spectrum of tissue elasticities, supporting its potential applicability across different pediatric developmental stages.

### 3.4. Mechanotransduction and Osteogenic Signaling

**Figure 4:**
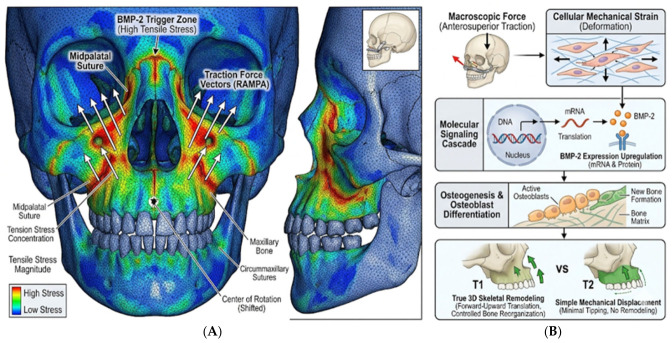
Mechanotransduction pathway and identification of the “BMP-2 Trigger Zone” during Right Angle Maxillary Protraction Appliance (RAMPA) therapy. (**A**) FEM Stress Distribution Map: Finite element analysis illustrating the concentration of tensile stress across the circummaxillary and midpalatal sutures. The highly stressed areas (warm colors) define the ‘BMP-2 Trigger Zone,’ where mechanical strain reaches physiological thresholds. (**B**) Biological Cascade Schematic: A conceptual representation of the mechanotherapeutic pathway. The macroscopic anterosuperior orthopedic forces are transduced into cellular mechanical strain, which actively upregulates Bone Morphogenetic Protein-2 (BMP-2) expression, thereby initiating molecular osteogenesis and true three-dimensional skeletal remodeling rather than simple mechanical displacement.

Beyond macroscopic skeletal displacement, the therapeutic efficacy of the RAMPA system is anchored in the principles of cellular mechanotransduction. Recent finite element analyses have specifically identified regions across the circummaxillary sutures where anterosuperior force vectors concentrate tensile stress, defining a theoretical “BMP-2 Trigger Zone” (Figure 4A). In these highly stressed regions, mechanical strain reaches the biological thresholds necessary to induce osteogenic signaling and the upregulation of Bone Morphogenetic Protein-2 (BMP-2), as conceptualized in the mechanotherapeutic pathway (Figure 4B). This suggests that RAMPA acts not merely as a physical expander, but as a mechanotherapeutic modulator that is hypothesized to trigger molecular signaling for active bone formation and remodeling at the sutural microenvironment.

## 4. Aerodynamic Reconfiguration and Fluid Mechanics (CFD)

The orthopedic expansion of the maxilla directly alters the internal geometry of the nasal airway. To understand how these skeletal changes may improve respiratory function, advanced Computational Fluid Dynamics (CFD), particularly two-phase air–mucus volume of fluid (VOF) simulations, have been employed.

### 4.1. Reduction of Airflow Resistance

CFD models were reconstructed from a representative study group of pediatric patients presenting with maxillary hypoplasia and constricted airways. These patient-specific CBCT data reveal that the RAMPA-induced widening of the nasal passages substantially alters internal aerodynamics. An expanded nasal cavity increases the cross-sectional area, reducing overall airflow resistance and stagnant airflow zones. The resulting acceleration in airflow velocity across the superior nasal meatus facilitates ventilation to reach the critical ostial pathways connecting the paranasal sinuses.

### 4.2. Two-Phase Fluid Dynamics and Mucus Clearance

**Figure 5:**
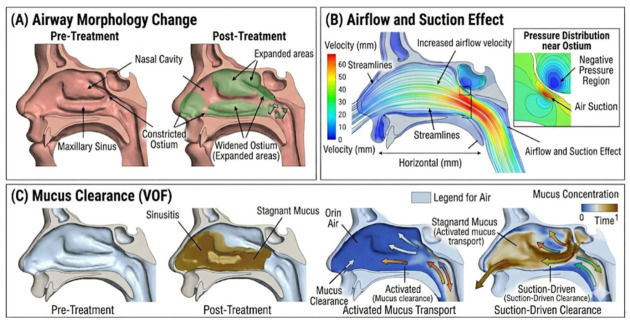
The Impact of Right Angle Maxillary Protraction Appliance (RAMPA) Therapy on Nasal Cavity and Sinus Aerodynamics and Mucus Clearance. (**A**) Airway Morphology Change: A comparison of sagittal skull models showing pre-treatment constricted nasal cavity and sinuses, and post-treatment expanded areas (highlighted in transparent green). (**B**) Airflow and Suction Effect: Visualization of improved aerodynamics using post-treatment geometry. Colored streamlines indicate expanded airway flow and increased airflow velocity, while the pressure distribution inset illustrates the formation of a negative pressure region near the ostium, resulting in an air suction effect into the sinus. (**C**) Mucus Clearance (VOF): A conceptual schematic illustration based on the theoretical dynamics of VOF two-phase flow. The top rows represent a sinusitis condition with stagnant mucus within a constricted sinus. The bottom rows demonstrate how the newly developed negative pressure mobilizes the mucus cluster, facilitating its clearance from the maxillary sinus into the nasal cavity via suction-driven forces.

Impaired mucus drainage is the primary pathophysiological driver of chronic rhinosinusitis and pediatric empyema. Using unsteady RANS turbulence models and non-Newtonian shear-thinning frameworks, two-phase CFD analyses have mapped the interaction between accelerated airflow and viscous mucus (Figure 5). The simulations demonstrated that the structural expansion achieved via RAMPA + sRME (Figure 5A) significantly enhances the “suction effect” near the sinus ostia (Figure 5B). This aerodynamic shift theoretically drives mucus expulsion from the maxillary and frontal sinuses, suggesting that RAMPA may act as a mechanotherapeutic intervention that facilitates the resolution of mucostasis without the immediate need for surgical or primary pharmacological intervention. However, it is important to note that these CFD simulations represent modeling-based hypotheses; definitive confirmation of mucostasis resolution requires direct clinical sinonasal outcomes, such as endoscopy, rhinomanometry, or mucociliary clearance testing.

Furthermore, to accurately capture the complex behavior of respiratory mucus, recent CFD simulations have successfully employed the Carreau–Yasuda rheological framework. While the comprehensive computational setup is detailed in our primary investigation [63], the core boundary conditions for the mucus phase in these simulations included a linear weighted ratio of density of 1000 kg/m^3^, surface tension of σ = 0.072 N/m, specific contact angle of 90° at the sinus ostium to accurately model mucosal adhesion. Additionally, the Carreau–Yasuda viscosity parameters (e.g., zero-shear viscosity of $\mu_{0}$ = 2.0 Pa·s, infinite-shear viscosity of $\mu_{\infty }$ = 7 × 10^−4^ Pa·s, and power-law index of *n* = 0.25, relaxation time λ = 40 s, and Yasuda parameter a = 2.0) were used for the simulation of hyperconcentrated, pathological mucus. These advanced two-phase air–mucus models demonstrate that the structural expansion achieved by RAMPA therapy significantly reduces wall shear stress (WSS) and improves overall airflow distribution. This optimized aerodynamics provides the critical physical conditions necessary to facilitate the transition of mucus from a stagnant, high-viscosity state to a mobilizable fluid state via shear-thinning effects. Consequently, this aerodynamic reconfiguration physically drives mucus expulsion from the sinuses (Figure 5C), indicating that RAMPA resolves mucostasis through sophisticated fluid-structure interactions.”

## 5. Clinical Outcomes and Volumetric Tracking

The translation of these computational predictions into clinical reality has been further investigated through large-cohort retrospective studies utilizing both traditional cephalometrics (Coben analysis) and advanced 3D CBCT volumetry.

### 5.1. Skeletal Growth and Coben Analysis

**Figure 6:**
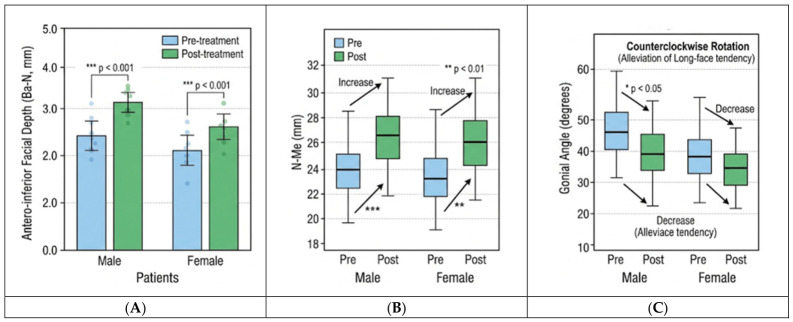
Statistical analysis of changes in facial skeletal morphology following Right Angle Maxillary Protraction Appliance (RAMPA) therapy using Coben analysis (*n* = 30). (**A**) Facial Depth (Ba-N, mm): Demonstrates a significant increase in post-treatment values across both male and female cohorts (*p* < 0.001). (**B**) Anterior Facial Height (N-Me, mm): Visualizes significant increases in N-Me values for both sexes (*p* < 0.01, *p* < 0.001), indicating controlled vertical growth. (**C**) Mandibular Angle (Gonial Angle, degrees): Reveals a significant decrease (*p* < 0.05) and counterclockwise rotation, specifically in the male cohort; the female group shows a downward trend with lower statistical significance.

In a retrospective cohort of 30 growing patients 30 growing patients (17 males, mean age 7.32 years; 13 females, mean age 8.34 years, treated over an average duration of 18 months), Coben analysis showed statistically significant increases in facial depth (Ba-N) and anterior facial height (N-Me) for both sexes following RAMPA therapy (Figure 6A,B). Notably, this period of treatment was associated with structural mandibular adaptation: male patients exhibited an average 1.47° decrease in the Gonial Angle (indicative of anterior rotation) (Figure 6C), while female patients demonstrated a significant increase in the maxillary depth ratio. These observational findings suggest the system’s potential to support 3D craniofacial balance in skeletal Class III profiles, although future controlled studies are necessary to definitively isolate the therapeutic effect from natural physiological growth.

### 5.2. Accelerated Sinonasal Volumetric Expansion

**Figure 7:**
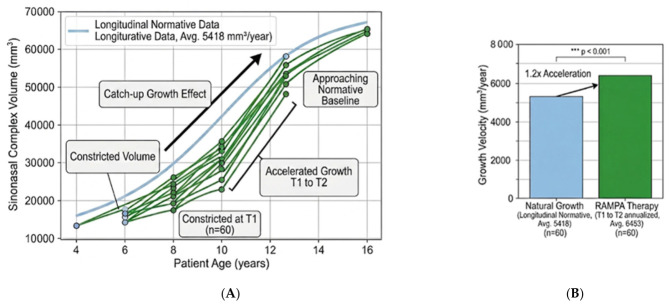
Volumetric growth velocity comparison of the sinonasal complex: Natural growth versus Right Angle Maxillary Protraction Appliance (RAMPA) therapy (*n* = 60). (**A**) Volumetric Growth Trajectory: Illustrates changes in sinonasal complex volume relative to patient age. Patients beginning treatment with pathologically constricted volumes (below the light-blue longitudinal normative baseline) exhibit a rapid “catch-up” growth trajectory (steep green lines) that converges toward normal levels. (**B**) Annual Volumetric Growth Velocity Comparison: A quantitative bar chart demonstrating that the annualized growth velocity under RAMPA therapy (avg. 6453 mm^3^/year) is significantly faster than the natural growth baseline (avg. 5418 mm^3^/year, derived from historical normative data [61,65]), achieving a 1.2-fold acceleration effect (*p* < 0.001).

A volumetric analysis of the complete sinonasal complex (nasal cavity and all four paranasal sinuses) in 60 pediatric patients indicated a 16.24% mean volume increase over an average treatment period of just 8.38 months. Furthermore, the annualized volumetric growth velocity observed during RAMPA therapy was approximately 1.2 times higher than the natural physiological growth baselines (Figure 7B). These normative baselines (averaging 5418 mm^3^/year) were derived from the pediatric volumetric data reported by Yamakawa et al. [65], which we utilized as a historical control cohort in our previous longitudinal study [61]. The control subjects from the normative data were cross-sectionally matched with the treatment group for age and sex; however, exact matching for skeletal maturity was limited by the retrospective nature of the available normative datasets. This observed “catch-up” growth trajectory (Figure 7A) suggests that RAMPA may be associated with an acceleration effect, potentially influencing the respiratory environment faster than natural maturation. However, because this comparison relies on historical normative data, the specific contributions of the appliance versus natural growth variation, selection bias, or segmentation variability cannot be definitively isolated without future prospective studies.

### 5.3. Differentiated Mechanisms: Clear vs. Opacified Sinuses

A key comparative study investigating RAMPA’s potential mechanotherapeutic effects differentiated patients based on baseline sinonasal health: those with clear paranasal sinuses (*n* = 26, mean age 6.6 years) versus those with pre-existing opacification or empyema (*n* = 20, mean age 6.8 years). The mean treatment duration for these combined cohorts was approximately 9 months. While both groups experienced an increase in upper airway volume following RAMPA therapy, the magnitude and hypothesized underlying mechanisms varied significantly.

- Clear Sinus Group (*n* = 26): Patients exhibited an 18% mean increase in sinonasal volume. Importantly, this increase positively correlated with treatment duration (r = 0.52, *p* < 0.05). This suggests a potential mechanism of gradual mechanically driven skeletal adaptation, where steady expansion may occur over time as a result of the continuous orthopedic force.
- Opacified Sinus Group (Empyema, *n* = 20): In contrast, patients presenting with chronic sinusitis or empyema exhibited a 61.2% mean increase in volume (*p* < 0.0001). Notably, this volume increase showed no significant correlation with treatment time (r = 0.08, *p* = 0.65). This lack of correlation suggests a potential duration-independent reduction of soft-tissue obstruction and mucosal edema.

**Figure 8:**
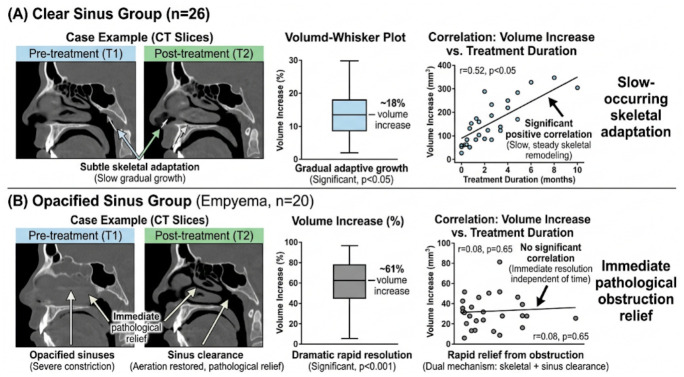
Differential mechanisms of airway expansion following RAMPA therapy based on baseline sinus status. (**A**) Clear Sinus Group (*n* = 26): Sagittal CT images (**Left**) show subtle skeletal adaptation. The box plot (**Middle**) indicates an ~18% volume increase, which strongly correlates with treatment duration (**Right**), highlighting a slow, steady remodeling process. (**B**) Opacified Sinus Group (*n* = 20): CT images (**Left**) demonstrate dramatic clearance of severely opacified sinuses. The box plot (**Middle**) reveals a massive ~61% volume surge. The lack of correlation with treatment duration (**Right**) emphasizes an immediate pathological obstruction relief independent of mechanical remodeling time.

These volumetric findings suggest that by potentially reducing chronic mucosal inflammation through improved aerodynamics, RAMPA therapy may function as a comprehensive mechanotherapeutic option that goes beyond simple skeletal expansion. However, confirming the exact clinical resolution of mucosal inflammation requires direct endoscopic or symptomatic validation. The clinical manifestations of these divergent patterns are detailed in Figure 8.

## 6. Discussion

This feature paper comprehensively synthesizes finite element modeling (FEM), computational fluid dynamics (CFD), and clinical CBCT data to explore the proposed multi-disciplinary mechanisms underlying RAMPA therapy. The synthesized evidence suggests that RAMPA may offer structural advantages over conventional mechanical expansion, potentially functioning as a “mechanotherapeutic modulator.” By conceptually bridging macroscopic orthopedic forces with hypothesized microscopic biophysical and molecular responses, this review proposes a theoretical framework linking initial mechanical intervention to the potential improvement of sinonasal homeostasis.

### 6.1. The Mechanotherapeutic Chain of Causality

The foundational mechanism of RAMPA therapy lies in its biomimetic approach to skeletal translation. Conventional therapies often induce rotational side effects that limit true three-dimensional airway expansion. In contrast, FEM studies reviewed herein suggest that RAMPA’s anterosuperior force vectors effectively mitigate the undesirable clockwise mandibular rotation (thereby promoting favorable counterclockwise mandibular rotation) while concentrating tensile stress directly across the circummaxillary sutures. Crucially, this targeted stress distribution reaches the mechanical thresholds hypothesized to form theoretical “BMP-2 Trigger Zones.” Within these zones, mechanical strain is transduced into osteogenic signaling, upregulating Bone Morphogenetic Protein-2 (BMP-2) and potentially catalyzing active, molecular-level bone remodeling.

The proposed skeletal remodeling initiated by this mechanotransduction profoundly alters the internal fluid dynamics of the sinonasal complex. CFD simulations utilizing the Carreau–Yasuda rheological framework indicate that structural expansion significantly reduces overall airflow resistance while optimizing Wall Shear Stress (WSS) distribution. Because respiratory mucus is a non-Newtonian fluid, the increased airflow velocity and enhanced suction effects generated by the expanded airway exert critical shear forces on stagnant mucus. This theoretically induces a “shear-thinning” effect, which may cause a biophysical breakdown in mucus viscosity. Consequently, highly viscous, trapped mucus could become fluidized and be actively expelled, suggesting that RAMPA has the potential to facilitate functional clearance through sophisticated fluid-structure interactions.

### 6.2. Clinical Significance of Pathophysiological Resolution

The clinical outcomes from large-cohort longitudinal analyses provide observational support for this multi-scale mechanotherapeutic framework. A key finding highlighted in this review is the differential response based on baseline sinonasal health. Patients with pre-existing empyema (opacified sinuses) exhibited a 61.2% volumetric increase, compared to a more gradual 18% increase in patients with clear sinuses. This contrast suggests a potential dual mechanism of action: in healthy airways, RAMPA appears to be associated with slow-occurring skeletal adaptation; in compromised airways, it may facilitate a duration-independent reduction of pathophysiological obstruction and mucosal edema. Furthermore, the overall 1.2-fold higher sinonasal growth velocity observed during treatment suggests that RAMPA has the potential to help guide constricted, pathological anatomies toward normative developmental baselines.

### 6.3. Limitations and Future Perspectives

While the integration of FEM, CFD, and clinical outcomes proposes a cohesive mechanotherapeutic framework for RAMPA therapy, several methodological limitations must be acknowledged.

First, computational models (FEM and CFD) are invaluable for generating mechanistic hypotheses and demonstrating biomechanical plausibility; however, they cannot intrinsically prove in vivo biological processes or definitive clinical mucus clearance. For example, while current FEM models provide predictive mapping of ‘BMP-2 Trigger Zones,’ direct in vivo quantification of molecular markers—such as BMP-2 levels in gingival crevicular fluid or inflammatory cytokines (e.g., TNF-α) in nasal secretions—remains a critical necessity to validate the cellular mechanotransduction pathways hypothesized in this review. Furthermore, incorporating patient-specific dynamic CFD models that account for varying degrees of mucosal inflammation will be necessary to refine our understanding of shear-thinning clearance mechanisms.

Second, the clinical evidence supporting this framework, including CBCT volumetric changes and Coben analyses, is primarily derived from retrospective cohorts. These observational studies face inherent limitations, including the lack of randomized control groups, potential selection bias, and the confounding effects of natural growth and maturation. While notable volumetric gains in empyema patients were observed, spontaneous sinus improvement, regression to the mean, and segmentation reliability cannot be entirely ruled out without rigorous, blinded assessments. Moreover, potential confounding factors—such as variations in patient compliance with the extraoral appliance and individual physiological growth spurts during the treatment period—must be carefully accounted for in future investigations.

Therefore, while current data suggest significant biomechanical potential, future well-designed prospective, randomized controlled trials with extended follow-up periods and molecular validation are essential to completely isolate the therapeutic effect and rigorously evaluate the long-term clinical efficacy of RAMPA therapy compared to conventional orthopedic appliances.

## 7. Conclusions

The integration of biomechanical engineering and clinical tracking suggests that the Right Angle Maxillary Protraction Appliance (RAMPA) may serve as a promising, biomimetic intervention for craniofacial hypoplasia. By maintaining controlled anterosuperior force vectors across varying suture stiffness levels, RAMPA aims to mitigate the rotational pitfalls of traditional appliances and is hypothesized to initiate sutural mechanotransduction, potentially stimulating molecular osteogenic signaling for true three-dimensional bone remodeling. More importantly, this mechanically guided skeletal remodeling is theorized to optimize internal aerodynamics—reducing airflow resistance, potentially facilitating shear-thinning mucus clearance, and yielding volumetric airway gains that are observed to exceed historical natural growth baselines. Ultimately, RAMPA therapy offers a comprehensive structural alternative to traditional dental alignment, providing a potential mechanotherapeutic option for pediatric craniofacial and respiratory compromise, though further prospective clinical validation remains necessary.

## Figures and Tables

**Table 1:** Summary of primary intraoral appliances used with RAMPA.

Intraoral Appliance	Design & Characteristics	Key Biomechanical & Clinical Effects with RAMPA
gHu-1 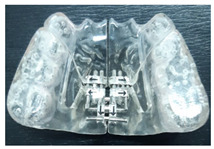	Semi-rapid maxillary expansion (sRME) device: Guides progressive lateral expansion of the palatal suture via screw activation.	Simultaneous expansion without interference: Anterosuperior protraction force of RAMPA and lateral expansion occur simultaneously without mechanical interference. Transformation of displacement vector: Transforms subtle postero-inferior displacement that may occur in the mid-palatal suture region into distinct anterosuperior lifting (FEM verified).
VomPress 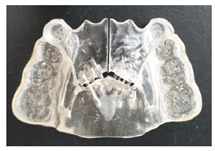	Space-creating appliance: Designed to physically expand intraoral volume to secure initial space for tongue positioning and airway.	Correction of cervical posture: As the airway opens through anterosuperior traction of the maxilla and securing space, it clinically induces distinct improvement in Straight Neck Problem and overall cervical posture. Effective improvement of anterior overjet and malocclusion.
Hybrid 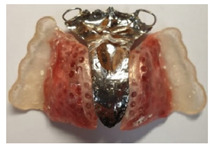	Novel Hybrid appliance (Multi-control type): Latest model combining advantages of existing devices to control three-dimensional movement of the maxilla more precisely.	Control of mandibular rotation: Induces counterclockwise rotation (−1.0 degree) and decrease in RAMUS angle (2.9 degrees) of the mandible to effectively alleviate Long-face Syndrome. Maximization of anterosuperior rotation: Demonstrates a synergistic effect maximizing anterosuperior rotation of the maxilla complex while minimizing downward displacement of the mid-palatal suture.

## Data Availability

The data presented in this study are available upon request from the corresponding author. The clinical dataset (CT scans) is not publicly available due to patient privacy and ethical restrictions.
